# Construction and characterization of centromeric plasmids for *Komagataella phaffii* using a color-based plasmid stability assay

**DOI:** 10.1371/journal.pone.0235532

**Published:** 2020-07-02

**Authors:** Luiza Cesca Piva, Janice Lisboa De Marco, Lidia Maria Pepe de Moraes, Viviane Castelo Branco Reis, Fernando Araripe Gonçalves Torres

**Affiliations:** Departamento de Biologia Celular, Bloco K, primeiro andar, Universidade de Brasília, Brasília, Brazil; Institut de Genetique et Microbiologie, FRANCE

## Abstract

The yeast *Komagataella phaffii* is widely used as a microbial host for heterologous protein production. However, molecular tools for this yeast are basically restricted to a few integrative and replicative plasmids. Four sequences that have recently been proposed as the *K*. *phaffii* centromeres could be used to develop a new class of mitotically stable vectors. In this work, we designed a color-based genetic assay to investigate plasmid stability in *K*. *phaffii* and constructed vectors bearing *K*. *phaffii* centromeres and the *ADE3* marker. These genetic tools were evaluated in terms of mitotic stability by transforming an *ade2/ade3* auxotrophic strain and regarding plasmid copy number by quantitative PCR (qPCR). Our results confirmed that the centromeric plasmids were maintained at low copy numbers as a result of typical chromosome-like segregation during cell division. These features, combined with *in vivo* assembly possibilities, prompt these plasmids as a new addition to the *K*. *phaffii* genetic toolbox.

## Introduction

*Komagataella phaffii* is a methylotrophic yeast of great industrial importance that has been used for more than 30 years as a heterologous protein production platform [1]. Its genome was firstly published in 2009 and has since then been refined and thoroughly studied [2, 3]. As a result, in addition to a protein factory, *K*. *phaffii* has also been widely considered as a platform for the production of chemicals, biopharmaceuticals, vitamins, and other molecules. However, the construction and regulation of new pathways demand complex molecular biology tools that are not yet readily available for this yeast [4].

*K*. *phaffii* genetic manipulation traditionally involves the use of shuttle vectors assembled in *Escherichia coli* and subsequently integrated in the yeast genome [5]. Recent studies have described the development of a wide range of genetic parts for use in this yeast, as well as new methods of plasmid assembly and transformation [6]. An alternative to integrative strategies is the use of replicative plasmids, which are usually based on the well-known autonomously replicating sequence 1 (ARS1) [1]. These plasmids may overcome some drawbacks such as the genetic instability in multi-copy strains, non-specific genomic integration, and different expression levels depending on the integration *locus* [7–9]. In addition, they present higher transformation efficiency when compared to integrative vectors and can be assembled by *in vivo* recombination, which eliminates the need for bacterial transformation [10, 11]. However, replicative plasmids show low mitotic stability when compared to integrative vectors and there are few vector options of this kind [12]. Stability problems can be circumvented by the construction of centromeric plasmids, which may provide proper segregation during mitosis. A greater mitotic stability, as well as low copy numbers, would allow stable and constant protein expression [13]. Considering that centromeric vectors can be assembled *in vivo*, allowing the cloning of large sequences (including whole metabolic pathways and regulatory regions) [14], the construction of such vectors would be of great value for *K*. *phaffii* strain development in the context of synthetic biology.

In most organisms, centromeres are typically surrounded by large heterochromatin sections [15]. Their structure ranges from simple “point” centromeres of only ~125 bp in *Saccharomyces cerevisiae* to epigenetic, sequence-independent centromeres such as those present in plants and animals. The reason for this phenomenon is that, for most eukaryotes, centromeres are maintained epigenetically and not genetically. Sequence homologies are rare in and between species, hindering the definition of a consensus sequence. In addition, some DNA regions can be centromeric or not depending on its function in previous cell cycles, which highlights the epigenetic nature of the centromere [16].

As for non-conventional yeasts, there are wide variations in centromere size and structure. *Candida glabrata* has centromeres that show some homology to the CDEI and CDEIII regions of *S*. *cerevisiae* while *Kuraishia capsulata* centromeres have 200-bp conserved sequences [17,18]. On the other hand, *Candida tropicalis*, *Schizosaccharomyces pombe*, and *Candida albicans* have regional centromeres named after their sizes, which range from 3 to 110 kb [19–21].

*K*. *phaffii* centromeres have recently been identified, bearing no sequence similarities to those of any other yeast [3]. Since centromere function relies strongly on its structure rather than on its sequence, a centromere-specific histone H3 variant (Cse4) was used in the search for centromeric regions in *K*. *phaffii*. A Cse4 homolog was identified in chromosome 2 and tagged with a fluorescence marker. The corresponding nuclear localization of the histone-DNA complex indicated a centromere pattern typical of budding yeasts [3]. Tridimensional conformation analysis followed the centromere clustering pattern observed in yeasts and narrowed down all four *K*. *phaffii* centromere locations to 20-kb windows [22].

Considering that a low transcription rate is typical of centromeric regions, RNA-seq analyses allowed researchers to pinpoint the putative centromeric locations for all four *K*. *phaffii* centromeres [3]. As is the case in *C*. *tropicalis* and *S*. pombe, *K*. *phaffii* centromeres are formed by inverted repeats. All four sequences have two inverted repeats of ~2.5 kb separated by a central segment of 800 to 1300 bp. Analyses of chromatin immunoprecipitation showed that the Cse4 histone bound preferably to the central region of the centromeres, but also along the inverted repeats [23].

*K*. *phaffii* centromeric sequences contain early replication peaks with autonomously replicating sequences, which is also observed in centromeres of other yeasts [24, 25]. According to recently published studies, there are native ARSs contained within centromeres 2 and 4. These comprise regions within the inverted repeats, as well as unique adjacent sequences [12, 23, 26].

In order to expand the molecular toolbox used in *K*. *phaffii* genetic manipulation, in this study we developed a genetic system based on an *ade2/ade3* auxotrophic strain and a replicative vector carrying the wild-type *ADE3* gene. Vectors carrying *K*. *phaffii* centromeres were then constructed and transformed to assess plasmid copy number and mitotic stability.

## Materials and methods

### Strains and media

DNA cloning was performed using chemically competent *Escherichia coli* XL-10 Gold (Agilent Technologies) grown in Luria-Bertani (LB) medium (5 g L^-1^ yeast extract, 10 g L^-1^ peptone and 10 g L^-1^ NaCl, pH 7.2). When needed, agar was added to a final concentration of 1.5%. When zeocin (25 μg mL^-1^) was used for bacterial antibiotic selection, the NaCl concentration was reduced to 5 g L^-1^.

*K*. *phaffii* strains were derived from X-33 (Invitrogen). The LA2 strain (*amd2 ade2*) was described in previous work [27]. Yeast was routinely grown in YPD medium (10 g L^-1^ yeast extract, 20 g L^-1^ peptone and 20 g L^-1^ glucose). Solid medium used 2% agar. Zeocin and geneticin, when used, were added at 100 μg mL^-1^ and 500 μg mL^-1^, respectively. Hygromycin B was used to a final concentration of 50 μg mL^-1^. MD medium used 0.34% Yeast Nitrogen Base, 1% (NH_4_)_2_SO_4_, 2% glucose, 0.00004% biotin, and 0.0002% adenine or 0.004% histidine, when needed.

### PCR

DNA was amplified using Invitrogen Platinum Taq DNA Polymerase (High Fidelity), Promega GoTaq Colorless Master Mix, or Sigma-Aldrich Accutaq LA DNA Polymerase. All primers used in this work are shown in Table 1.

**Table 1:** Primers used in this work.

Primer	Sequence	Enzyme
ADE3up-F	GATAAGCTTGATATCGAATTCCTGCAGCCCCCCGGGACGTAATGGAATAACTGCTGAC	*Sma*I
ADE3up-R	GAAGTTATGGATCCTACGAGGTAATTGAAGGCTCAC	
ADE3lox-F	CTTCAATTACCTCGTAGGATCCATAACTTCGTATAATG	
ADE3lox-R	CAATCTCTCCCTTGTCATCGGATCCATAACTTCGTATAG	
ADE3dw-F	GAAGTTATGGATCCGATGACAAGGGAGAGATTGAAG	
ADE3dw-R	GGTGGCGGCCGCTCTAGAACTAGTGGATCCCCCCCCGGGTGCAATGTACTGTTGAGTAGG	*Sma*I
ADE3conF	GGGGACCGGAGGTAAAAGAC	
ADE3conR	GTTGGAATAATTGCATGGTCTG	
MUT1(Hpa)	CCATTGACATGGTAAACAGTTGGA	
MUT2(Bam)	GAACACTGGGATCTTGGTTGAGG	
Cen1-F	GAGTTTAAACGGATCCACGAAGCAATGGATAGGCACT	*Bam*HI
Cen1-R	CCGCGAATTCGGATCCTGAAGTCTTTCAGAGAGGAGCA	*Bam*HI
Cen1c-F	CAAGTATGCGTGATCCCAGGT	
Cen1c-R	TACGAATTGTGGGGCTCTGT	
Cen2-F	GAGTTTAAACGGATCCATCTCCGTTGATACTCCCAAC	*Bam*HI
Cen2-R	CCGCGAATTCGGATCCATCGACAAGCAGAACACTAAG	*Bam*HI
Cen2c-F	GAATGGAGGTGCTGGTGGTTA	
Cen2c-R	TGTAATGCTCGCTGGTGAGT	
Cen3-F	GAGTTTAAACGGATCCAAGTGGTACACCAGTCAGCG	*Bam*HI
Cen3-R	CCGCGAATTCGGATCCTCAGTATTCAACTGCAACTGC	*Bam*HI
Cen3c-F	TCAGCCGAATACCCACACTT	
Cen3c-R	TCAGCCGTCAGCGAAATGAT	
Cen4-F	GAGTTTAAACGGATCCCAAACGCACCGTCTTGTTCA	*Bam*HI
Cen4-R	CCGCGAATTCGGATCCAATTGATGTAGACGAGCAGC	*Bam*HI
Cen4c-F	TCAAGAATCGTACTGGCACCT	
Cen4c-R	CAAGCTCGTGAGATGGGATGT	
Cen370-F	CGCTCAGTGGAACGAAAACTCACGTTAAGGGATTTGGTCATGAGATCAGATCTAACATCCAAAGACGAAAGGTTGAATGAGTTTAAACGGATCCAAGTGG	
Cen370-R	GCTGGCCCTCTCTTCCCAGCTCACGAATCAGATCCTAAGTCCTACTCAACAGTACATTGCAGCGGCCGCGTTTAAACGAATTCGGATCCTCAGTATTCAA	
qZEO-F	CGACGTGACCCTGTTCATCA	
qZEO-R	TGGACACGACCTCCGACCA	
qHIS-F	GTGTATCCTGGCTTGGCATCT	
qHIS-R	GCCAAGTACGGTGTGACGTT	

Restriction sites are underlined.

### DNA manipulation

All basic DNA manipulation and analyses were performed as previously described [28]. Restriction digestion was performed in accordance to the manufacturer instructions (New England Biolabs), as well as vector dephosphorylation with Shrimp Alkaline Phosphatase (Promega), and ligation with T4 DNA Ligase (USB). The In-Fusion Cloning Kit (Clontech) was used for *in vitro* assembly of plasmids. Site-directed mutagenesis was performed using the Transformer Site-Directed Mutagenesis Kit (Clontech). PCR and gel purification used the Promega Wizard SV Gel and PCR Clean-Up System.

### qPCR

Three colonies harboring each of the zeocin resistance plasmids were grown to an OD_600_ (optical density measured at 600 nm) of 1 in 10 mL of YPD containing zeocin while LA3 was grown in 10 mL of YPD. Cells were collected by centrifugation at 2000 × *g* for 5 min. The cell pellet was resuspended with 1 mL 0.25% sodium dodecyl sulfate (SDS) and incubated at 98°C for 8 min according to previous work [26]. Finally, cellular debris was removed by centrifugation and the DNA was diluted 10-fold in nuclease-free water before the qPCR reactions.

qPCR reactions used primers qZEO-F and qZEO-R for plasmid quantification and qHIS-F and qHIS-R as an internal single-copy control. The assays used iTaq Universal SYBR Green Supermix (Bio-Rad) in a Rotor-Gene Q (Qiagen) thermal cycler in technical triplicates. The analysis used the absolute quantification method and standard curves that ranged from 1x10^3^ to 1x10^7^ copies of the gene of interest. pPIC9 (Invitrogen) and pPICH linearized plasmids were used for the construction of standard curves.

### Yeast transformation

*K*. *phaffii* was electroporated following two different protocols. For integrative cassettes, we followed the *Pichia* Expression Kit protocol (Invitrogen), and when using replicative plasmids, we proceeded as described previously [29].

### Construction of an *ade2 ade3* strain for color-based stability assays

Strain LA2 [27] was transformed with an *ADE3* deletion cassette and had the marker recycled before transformation with the centromeric plasmids. The construction of this deletion cassette used PCR reactions assembled by an In-Fusion cloning reaction. Briefly, primers ADE3up-F and R, ADE3dw-F and R were used for PCR amplification of 491 bp and 582 bp, respectively, of the *K*. *phaffii* genome. These sequences were used for directing the homologous recombination and substitution of the complete *ADE3* coding sequence. Meanwhile, primers ADE3lox-F and ADE3lox-R were used in the amplification of the *kanR* geneticin resistance cassette from plasmid pGKL [30]. All three PCR fragments were assembled and cloned into pBluescript II SK^+^ linearized with *Sma*I. A final PCR reaction using primers ADE3up-F and ADE3dw-R amplified the whole deletion cassette, which was used in the transformation of *K*. *phaffii* LA2. Clones were selected on YPD containing geneticin.

The resulting *ade2 ade3Δ*::*kanR* strain was later transformed with pYRCre2 [31] and clones were selected on YPD supplied with hygromycin B. This step promoted a Cre-mediated excision of the *kanR* cassette, eliminating geneticin resistance. After PCR confirmation of marker recycling using primers ADE3conF and ADE3conR, the resulting strain was plated on non-selective YPD medium and lost the pYRCre2 plasmid. The final strain was named LA3. Table 2 summarizes the strains used and constructed in this work.

**Table 2:** *K*. *phaffii* strains used in this work.

*K*. *phaffii* Strain	Genotype	Reference
X-33	Wild-type	Invitrogen
LA2	*amd2 ade2*	30
LA2*Δade3*	*amd2 ade2 ade3Δ*::*kanR*	This work
LA3	*amd2 ade2 ade3*	This work

### Construction of centromeric plasmids containing *ADE3*

Plasmid pPICH [27], derived from pPICHOLI (MoBiTec), contains the *K*. *phaffii* ARS1 replicating sequence [1]. This sequence is originally located on *K*. *phaffii* GS115 chromosome 2, coordinates 413701–413856 [2]. pPICH was digested with *Not*I for cloning of the *K*. *phaffii* native *ADE3* gene. The complete gene was amplified from *K*. *phaffii* X-33 DNA using primers ADE3up-F and ADE3dw-R. This PCR reaction amplified 4141 bp from *K*. *phaffii* chromosome 4, coordinates 477160–481331, containing coding sequences for the Ade3 domains (formate-tetrahydrofolate ligase, methylene-tetrahydrofolate dehydrogenase, and methenyl tetrahydrofolate cyclohydrolase) and at least 500 bp of adjacent sequences that contained the native promoter and terminator elements [2]. This amplicon was then submitted to restriction digestion with *Not*I. After vector dephosphorylation, fragments were ligated and transformed into *E*. *coli* XL-10 Gold. One positive clone was then submitted to site-directed mutagenesis using primers Mut1(Hpa) and Mut2(Bam) for removing the *Bam*HI restriction site present within the *ADE3* coding sequence. The final plasmid containing ARS1, the *Sh ble* resistance marker, and *ADE3* was named pPICH-ADE3. The maps for pPICH-ADE3 and all centromeric plasmids constructed in this work are present on S1 Fig.

**Fig 1:**
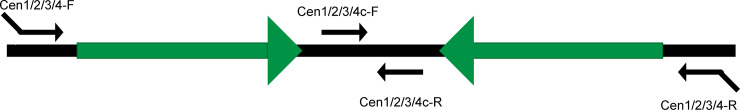
Strategy for the amplification of *K*. *phaffii* centromeres. Schematic representation of a typical *K*. *phaffii* centromere. Inverted repeats are represented by green arrows. Primer annealing regions are shown by black arrows.

pPICH-ADE3 was digested with *Bam*HI for the cloning of all four *K*. *phaffii* centromeres. Since the amplification of entire centromeric regions revealed to be extremely difficult, we designed a strategy to amplify centromeres in halves in order to reduce fragment size and avoid primer annealing inside the inverted repeats (Fig 1). The amplified fragments exhibited overlapping regions in their ends that would allow recombination between each other and with vector pPICH-ADE3.

Centromeric primer sequences were designed using *K*. *phaffii* GS115 genome sequence as reference [2]. The amplified regions corresponded to the following chromosomal coordinates: chromosome 1 position 1401429–1406917 (5488 bp), chromosome 2 position 1543739–1550657 (6918 bp), chromosome 3 position 2204800–2211493 (6693 bp), and chromosome 4 position 1703369–1709958 (6589 bp). Primers Cen1/2/3/4-F and Cen1/2/3/4c-R amplified the first inverted repeat of each centromere while primers Cen1/2/3/4c-F and Cen1/2/3/4-R amplified the other half of the sequences. In order to promote *in vitro*/*in vivo* assembly, the amplicons had ~80 bp homology between each other on one end and 15 bp with pPICH-ADE3 on the other end.

First, we attempted an In-Fusion cloning reaction for each of the four centromeres using the linearized pPICH-ADE3 and two PCR fragments. Plasmids were extracted from *E*. *coli* colonies and analyzed by restriction digestion. Centromeres 1, 2, and 4 were successfully assembled and cloned using this strategy. Centromere 3 did not yield any *E*. *coli* clones following the In-Fusion reaction; we then proceeded to an *in vivo* assembly strategy. Primers Cen370-F and Cen3c-R; Cen3c-F and Cen370-R amplified both inverted repeats adding 70 bp of homologous sequences between the fragments and pPICH-ADE3. Finally, we transformed *K*. *phaffii* LA3 using the linearized vector and both centromeric fragments, using 85 bp of homology for directing recombination. Clones were selected on YPD supplied with zeocin. However, none of the obtained clones presented the assembled plasmid as it was expected and this centromeric sequence was not used in further analyses.

The resulting plasmids were named pPICH-CEN1, pPICH-CEN2, and pPICH-CEN4 and their maps are available in S1B Fig. All plasmids were transformed into *K*. *phaffii* LA3 for subsequent stability and quantification assays.

### Stability analysis

Three colonies of the LA3 strain transformed with each of the three centromeric plasmids or pPICH-ADE3 were grown in 20 mL of YPD for 16 hours at 28°C and 200 rpm. These cultures were then inoculated in 20 mL of YPD to an initial OD_600_ of 0.1. After 24 h of growth under the same conditions, the cultures were inoculated in new flasks containing 20 mL of fresh YPD to an OD of 0.1. This procedure was repeated every 24 h until reaching 144 h of growth. At 96 and 144 h of growth, cells were diluted 10^4^-fold and 100 μL of this dilution were plated on YPD. Plates were incubated at 30°C for 72 h. After incubation, the numbers of red and white colonies obtained in each plate were registered and the results were analyzed with an unpaired *t*-test with Welch’s correction.

## Results and discussion

### Construction of *K*. *phaffi* LA3, an *ade2 ade3* double auxotrophic strain

In yeast, the adenine synthesis pathway is used as a tool for auxotrophic selection, gene copy number indication, and for plasmid stability analyses [32]. Many genes from this pathway have been deleted in *S*. *cerevisiae* in order to create auxotrophic strains, while in *K*. *phaffii* studies have only focused on *ADE1* and *ADE2* [33, 34]. *K*. *phaffii* LA2, a mutant *ade2* strain [27], was used as a starting point for the construction of a strain that would allow plasmid stability verification. The deletion of the *ADE2* gene results in cells that are auxotrophic for adenine and accumulate a red intermediate [32]; the additional deletion of genes located upstream in the adenine synthesis pathway, such as *ADE1* or *ADE3*, should prevent the formation of such pigment [33].

**Fig 2:**
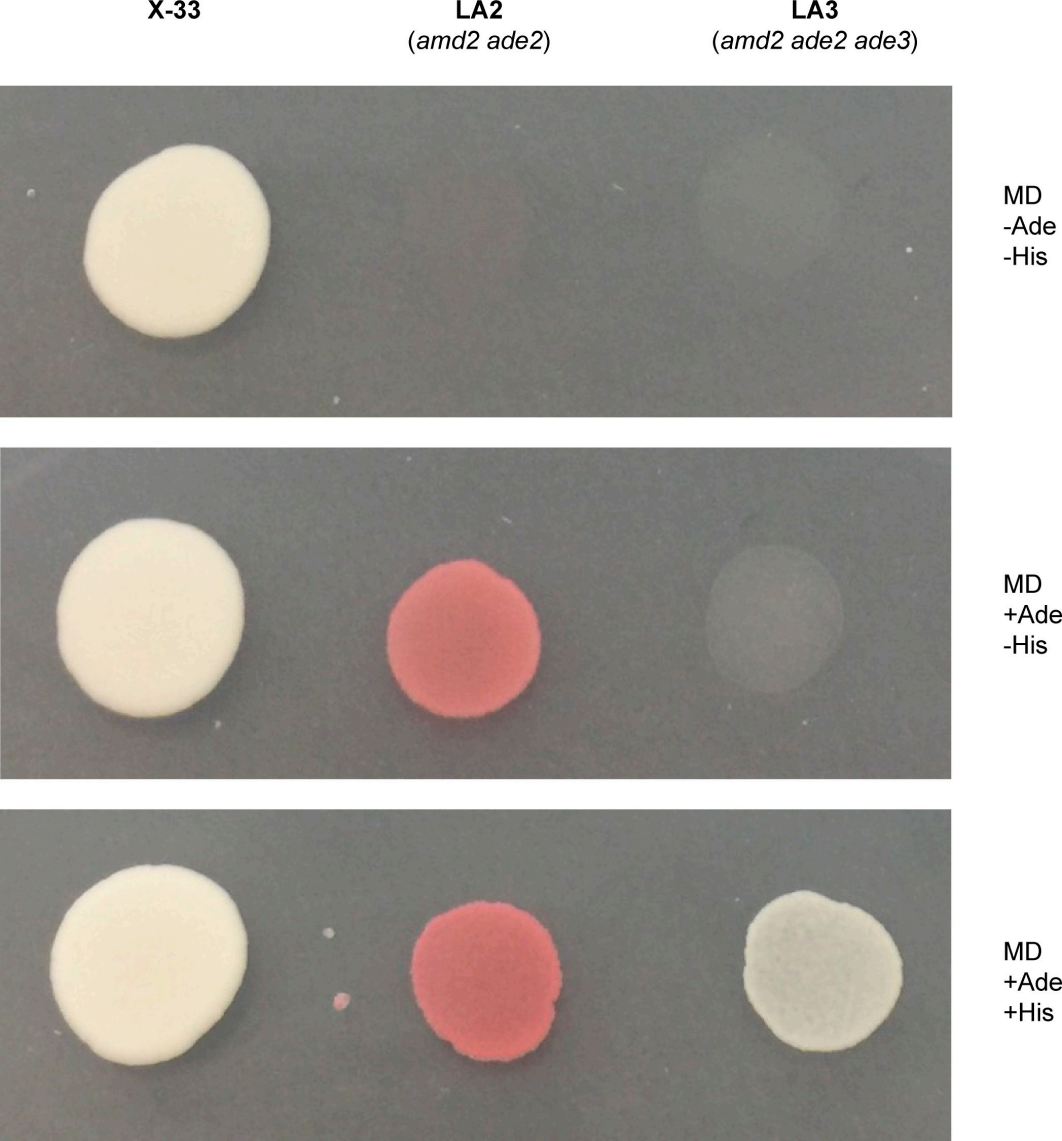
Strain phenotypic analysis on defined media. *K*. *phaffii* X-33 (wild-type), LA2 (*amd2 ade2*), and LA3 (*amd2 ade2 ade3*) cultures were spotted on a single plate of MD medium with adenine (Ade) or histidine (His) supplementation.

As expected, the *ADE3* deletion performed in the LA2 strain resulted in white colonies (Fig 2). In *S*. *cerevisiae*, this gene knock-out has regulatory effects in the histidine synthesis pathway [35]. Consequently, *ade2 ade3* strains are not only auxotrophic for adenine, but also for histidine. In order to verify if this phenotype was also true for *K*. *phaffii*, we plated strains X-33, LA2 (*amd2 ade2*), and LA3 (*amd2 ade2 ade3*) on minimal dextrose (MD) medium. This was performed using MD medium without supplementation, MD medium with adenine, or MD medium with both adenine and histidine supplementation. The growth and colony color of each strain were compared on the different medium compositions (Fig 2) and the LA3 strain displayed the expected histidine auxotrophy phenotype, showing that the adenine-histidine pathways in *K*. *phaffii* and *S*. *cerevisiae* have common characteristics.

### Plasmid stability

In order to assess plasmid stability, we first constructed plasmid pPICH-ADE3, carrying the wild-type *ADE3* gene (S1A Fig). When transformed with pPICH-ADE3, LA3 colonies (originally white) should become red and any changes in colony color would allow a simple screening of plasmid loss [32]. Although adenine auxotrophy has been explored for other purposes [34], this particular color-based system has not yet been used for measuring plasmid stability in *K*. *phaffii*.

pPICH-ADE3 was then used for cloning *K*. *phaffii* centromeres in its unique *Bam*HI site. Despite several attempts, we were unable to clone the centromere present in chromosome 3, which was excluded from our analysis. We speculate that its repetitive motifs, which were unlike those present in the other *K*. *phaffii* centromeres, rendered it extremely unstable in *E*. *coli*. Therefore, we proceeded with our analyses using centromeric plasmids that contained centromeres 1, 2, and 4 (pPICH-CEN1, pPICH-CEN2, and pPICH-CEN4).

**Fig 3:**
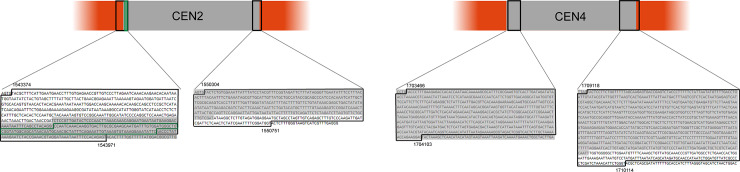
Relative positions and genomic coordinates of ARS sequences around centromeres 2 and 4 of *K*. *phaffii*. Regions in gray represent the centromeric sequences amplified in this work. Black rectangles indicate the identified ARS sequences [12], while the green rectangle indicates the 111-bp sequence already tested *in vivo* [26].

Centromeric sequences are known as early replication regions and recently published studies have proposed that there are native ARS cores contained within *K*. *phaffii* centromeres 2 and 4 [12, 23]. These ARSs were identified by sequencing methods, and Fig 3 illustrates their positions within and around the centromeres. A 111-bp fragment present in the beginning of centromere 2 has also been tested *in vivo* for its replicative activity. This sequence, when cloned in an ARS-less plasmid, was able to ensure plasmid replication and provided small colonies in *K*. *phaffii* transformation [26].

In chromosome 2, the proposed ARS core sequences are located on coordinates 1543374–1543971 (597 bp) and 1550304–1550751 (447 bp). These were partially amplified in this work, containing 232 and 354 bp, respectively. Chromosome 4 has an ARS core on coordinates 1703466–1704103 (637 bp), which was fully amplified, as well as a partially amplified one (840 bp) on coordinates 1709118–1710114 (996 bp total). All these ARS sequences were classified by Liachko and colleagues as AT-ARS, meaning that they do not contain the proposed GC-rich GC-ACS motif and thus resemble those of other budding yeasts like *S*. *cerevisiae* [12].

The LA3 strain was individually transformed with pPICH-ADE3 and centromeric plasmids pPICH-CEN1, 2, and 4. One clone of each construction was verified for autonomous replication by plasmid rescue in *E*. *coli*, and restriction digestion with *Not*I confirmed vector integrity (S2 Fig). The transformation efficiency obtained with plasmid pPICH-ADE3 was higher than those obtained with the centromeric plasmids (S1 Table).

**Fig 4:**
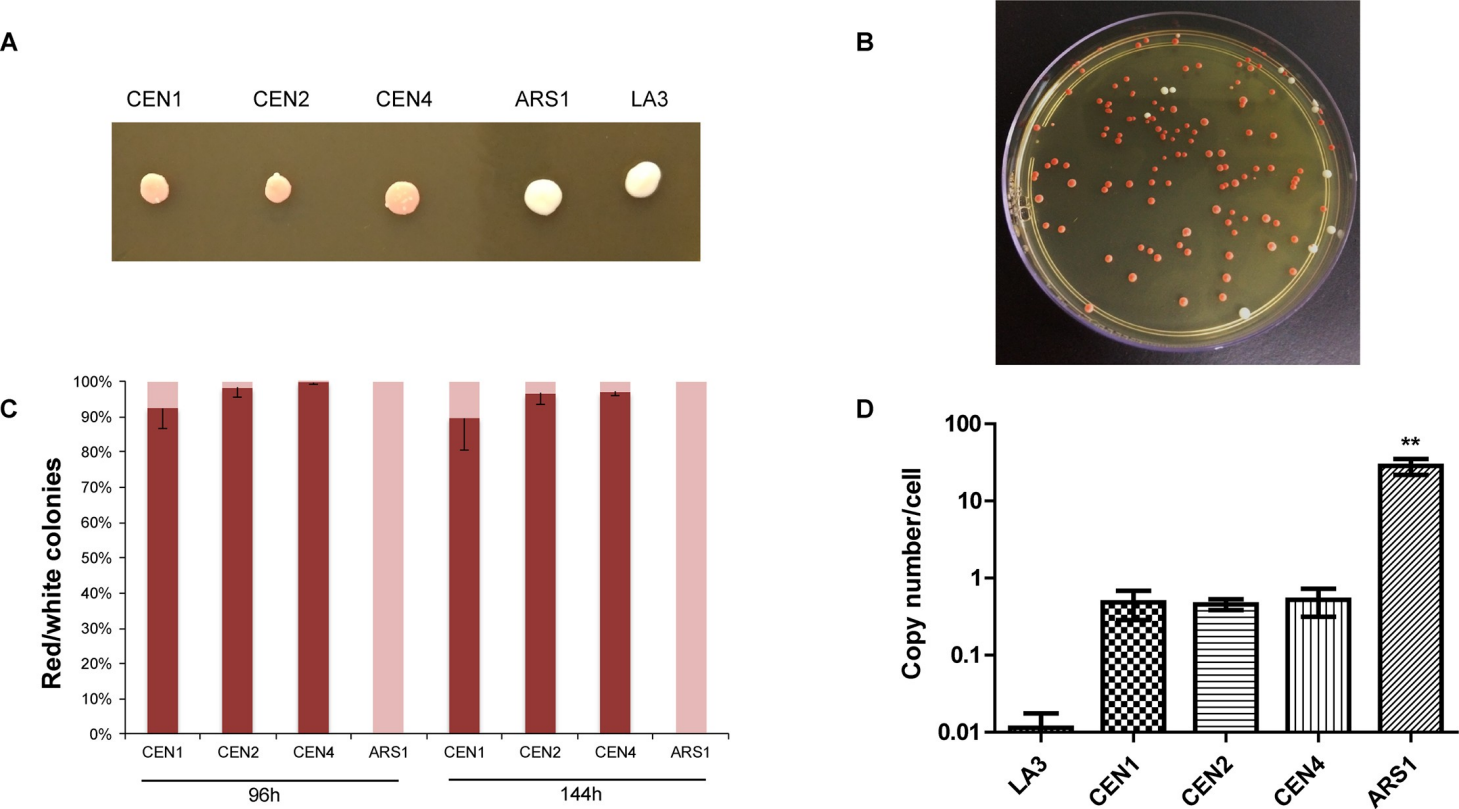
Plasmid stability and copy number analyses of the LA3 strain transformed with pPICH-CEN1 (CEN1), pPICH-CEN2 (CEN2), pPICH-CEN4 (CEN4), and pPICH-ADE3 (ARS1). **(A)** Representative colonies of each of the transformations and of the LA3 strain on YPD medium. **(B)** Plate representing a typical result of the plasmid stability analysis. Aliquots of three liquid cultures for each plasmid construction were collected and diluted after 96 and 144 h of growth; the dilutions were plated on YPD medium. **(C)** Proportions between red and white colonies obtained at each timepoint. Red colonies obtained in the plasmid stability plates are represented by red portions of the bars; light pink bars represent white colonies. Statistical analyses comparing the centromeric plasmids with each other were performed through unpaired t-tests with Welch’s correction for unequal variances using GraphPad Prism 5. Error bars represent the standard deviation of the mean (n = 3 biological replicates). **(D)** Plasmid copy number determination by quantitative PCR (qPCR). LA3 was used as negative control. Statistical analyses comparing each of the centromeric plasmids with the replicative vector were performed through unpaired t-tests with Welch’s correction for unequal variances using GraphPad Prism 5 (p<0.005). Error bars depict the standard deviation of the mean (n = 3 biological replicates).

Plasmid stability was firstly verified by colony color in non-selective medium (Fig 4A). When plated on yeast extract-peptone-dextrose (YPD) medium, colonies transformed with pPICH-ADE3 lost their red color rapidly and presented a white coloration; this result was consistent with plasmid instability. In contrast, strains transformed with the centromeric plasmids presented uniform pink colonies.

Further examination of the stability of centromeric plasmids was performed by growing cells in liquid YPD medium for 144 h. These cultures were diluted and plated on non-selective medium, and finally the red and white colonies obtained in each plate were counted and compared between each construction (Fig 4B and 4C). LA3 strain transformed with pPICH-ADE3 did not yield red colonies in any growth period, indicating that the plasmid was mitotically unstable. Conversely, all centromeric plasmids mostly presented red colonies, indicating a higher mitotic stability in comparison to pPICH-ADE3.

Yeast centromeric plasmids knowingly have a higher mitotic stability under non-selective conditions in comparison to common replicative vectors. This happens because they are equally segregated between daughter cells and therefore provide a uniform culture of cells containing the plasmid [32]. A centromeric vector containing *K*. *phaffii* CEN2 has been constructed and presented an enhanced stability when compared to a replicative plasmid [26]. In addition to *K*. *phaffii* and *S*. *cerevisiae*, centromeric plasmids have been developed for other yeasts such as *S*. *pombe*, *C*. *glabrata*, and *Scheffersomyces stipitis*, and in all cases presented enhanced stability under non-selective conditions [13, 18].

A striking difference between pPICH-CEN1 and the other centromeric plasmids is the absence of an ARS core within the centromeric region of the former. In addition, it has been shown that *K*. *phaffii* ARS elements may have different effects on plasmid stability [12]. Considering that our analyses evidenced a comparable mitotic stability among all centromeric plasmids, including those containing ARSs within the cloned centromeres, the role, if any, of these ARS cores in *K*. *phaffii* plasmid stability remains unclear. Future studies should focus on further characterizing the centromeric plasmids, especially pPICH-CEN1, along with different ARSs, in order to analyze the effects on plasmid stability.

### Plasmid copy number

Yeast replicative plasmids are normally replicated but are unevenly distributed between daughter cells, which creates cells with either multiple copies of the plasmid or none at all [32]. Under selective conditions, cells lacking the plasmid are unable to survive and the result is a population of multi-copy plasmid-containing cells. The construction of centromeric plasmids should provide better plasmid segregation and stability, allowing cells to maintain a low and stable plasmid copy number during yeast growth [36]. In this study, plasmid copy number was assessed by qPCR after three colonies of LA3 transformed with each centromeric plasmid were grown in YPD medium containing zeocin. The results were compared to those of the LA3 strain transformed with pPICH-ADE3 and grown in selective medium. The LA3 strain was used as control and grown in YPD medium.

qPCR results (Fig 4D) indicate that the strain transformed with centromeric plasmids carried less than one copy per cell, while the replicative plasmid was present at approximately 30 copies per cell. The difference in plasmid copy number between the replicative vector and each of the centromeric vectors was significant according to an unpaired *t*-test with Welch’s correction (p<0.005). This result illustrates the expected segregation pattern described above for growth in selective conditions and, together with the mitotic stability analysis, provides a clear picture of *K*. *phaffii* genetic manipulation using centromeric plasmids.

*S*. *cerevisiae* centromeric plasmids, in comparison to plasmids bearing the 2 micron sequence, presented the same difference in copy numbers when using auxotrophic markers. However, when the *kanMX* G418 resistance marker was used, the plasmid copy numbers did not differ between centromeric and replicative plasmids [37]. This indicates that factors other than the type of replication origin can influence plasmid copy numbers. In a previous study, *K*. *phaffii* transformed with a plasmid containing centromere 2 was analyzed regarding plasmid copy number and compared to both a replicative plasmid and an integrative strategy [26]. Results exhibited a low number of plasmids per cell in all strategies, which does not correspond to our observation. This difference may be related to the different strains, culture conditions, or plasmid constructions used in the two studies. Although both plasmids used the ARS1 replicative sequence and the zeocin resistance marker, pPICH-CEN1, 2, and 4 contained the *ADE3* gene while the previously reported centromeric plasmid carried an *EGFP* reporter gene.

Altogether, our results indicate that centromeric plasmids could be employed as a new tool for the genetic manipulation of *K*. *phaffii*. These plasmids were maintained for long periods in non-selective medium, indicating that yeast growth could be performed without any form of selective pressure. The low copy numbers exhibited by the centromeric plasmids characterize a stable and homogeneous culture that could provide reliable expression results. Finally, their structure as a circular molecule allows *in vivo* plasmid assembly with relatively short homologous sequences when compared to genome integration, where sequences have to be much longer for directing homologous recombination. A simpler assembly may enable the construction of larger and more sophisticated vectors such as yeast artificial chromosomes (YACs), whose stability features may also be analyzed by the color-based assay described in this work.

## Supporting information

S1 Fig **(A) Map of vector pPICH-ADE3.** ARS1 was used as the autonomously replicating sequence and *Sh ble* was used as the zeocin resistance marker. The *Not*I site was used for cloning of the *K*. *phaffii ADE3* gene. Images were generated using SnapGene 5.0.7. **(B) Maps of the centromeric plasmids constructed in this study.** Plasmids pPICH-CEN1, pPICH-CEN2, and pPICH-CEN4. Images were generated using SnapGene 5.0.7.
(PDF)

S2 Fig **(**A) Restriction analysis of recovered plasmids pPICH-CEN1, 2, and 4. Plasmids were digested with *Not*I and analyzed on 1% agarose gel. All digested plasmids yielded a common 4.2 kb band that represents the *ADE3* gene. The sizes of the upper bands represent the sum of individual centromeres and other common vector sequences (CEN1 = 7.6 kb; CEN2 = 9.0 kb; CEN4 = 8.7 kb). The upper band on pPICH-CEN1-*Not*I represents a partial digestion. M: 1 kb Plus DNA Ladder (Thermo Fisher Scientific). (B) Restriction analysis of pPICH-ADE3. The plasmid was digested with *Not*I and analyzed on 1% agarose gel. The 4.2-kb band represents the *ADE3* gene, and the 2.2-kb band represents the selection marker and *E*. *coli* sequences. M: 1kb Ladder Plus (Sinapse Inc).
(PDF)

S3 Fig Maps of the centromeric plasmids constructed in this study. Plasmids pPICH-CEN1, pPICH-CEN2, and pPICH-CEN4. Images were generated using SnapGene 5.0.7.
(PDF)

S1 Table Transformation efficiencies obtained in 3 transformation events with pPICH-ADE3 and pPICH-CEN1-4. (PDF)
