# Supplementary material for: Construction and characterization of centromeric plasmids for Komagataella phaffii using a color-based plasmid stability assay
Source: PLoS One. 2020 Jul 2;15(7):e0235532. doi: 10.1371/journal.pone.0235532 (PMC7332064; doi:10.1371/journal.pone.0235532)
Supplement: S1 Fig — (A) Map of vector pPICH-ADE3. ARS1 was used as the autonomously replicating sequence and Sh ble was used as the zeocin resistance marker. The NotI site was used for cloning of the K. phaffii ADE3 gene. Images were generated using SnapGene 5.0.7. (B) Maps of the centromeric plasmids constructed in this study. Plasmids pPICH-CEN1, pPICH-CEN2, and pPICH-CEN4. Images were generated using SnapGene 5.0.7. (PDF) [file pone.0235532.s001.pdf]

S1 Fig. (A) Map of vector pPICH-ADE3. ARS1 was used as the autonomously replicating sequence and *Sh ble* was used as the zeocin resistance marker. The *Not*I site was used for cloning the *K. phaffii ADE3* gene. Images were generated using SnapGene 5.0.7.

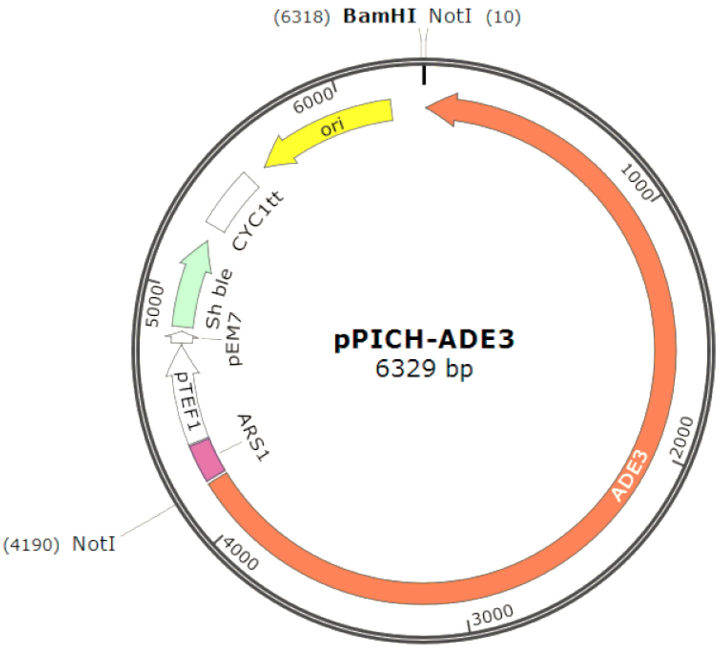

(B) Maps of the centromeric plasmids constructed in this study. Plasmids pPICH-CEN1, pPICH-CEN2, and pPICH-CEN4. Images were generated using SnapGene 5.0.7.

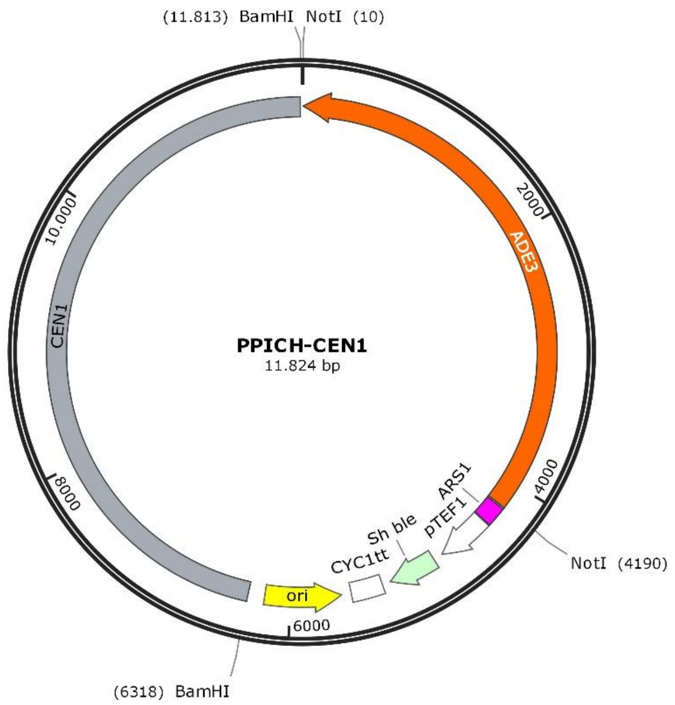

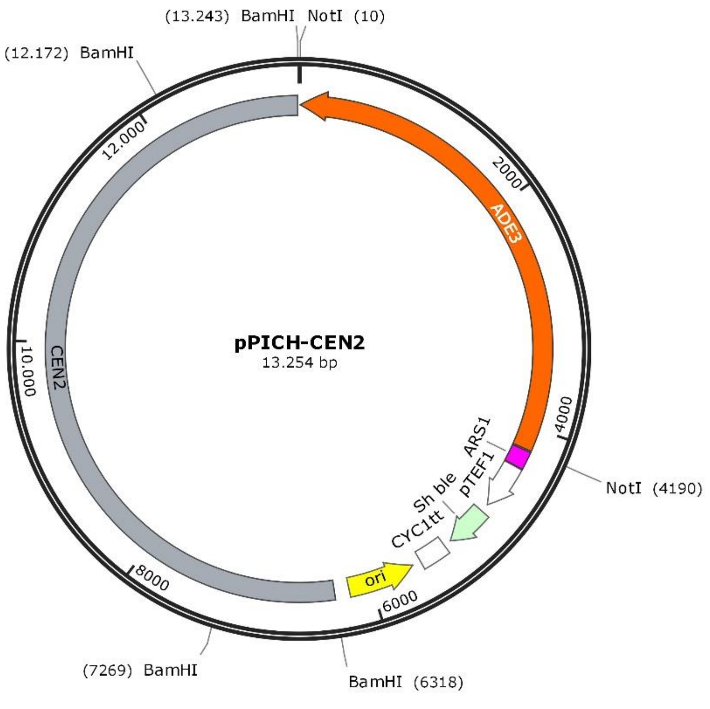

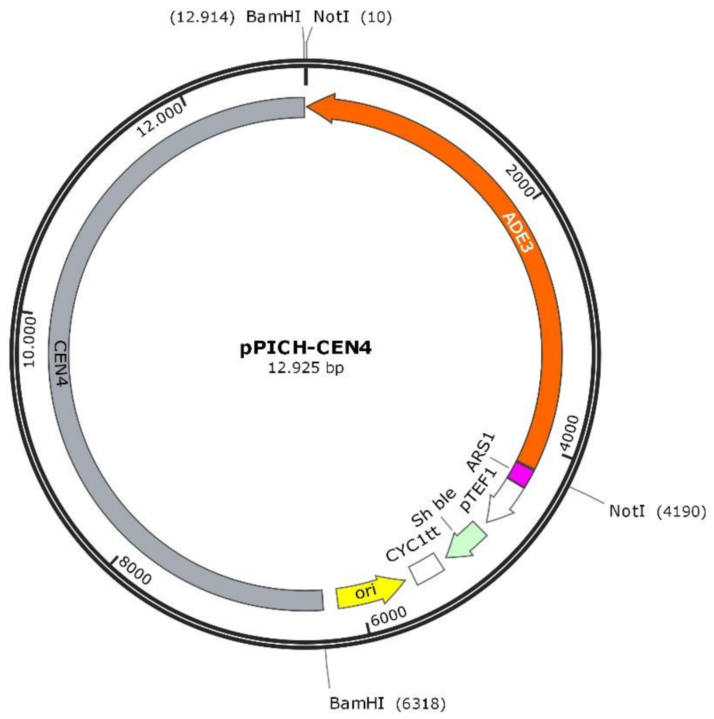
(12.914) BamHI NotI (10)
12.000
2000
ADE3
pPICH-CEN4
12.925 bp
10.000
CEN4
4000
ARS1
NotI (4190)
pTEF1
Sh ble
CYC1tt
8000
ori
6000
BamHI (6318)
