# Supplementary material for: Construction and characterization of centromeric plasmids for Komagataella phaffii using a color-based plasmid stability assay
Source: PLoS One. 2020 Jul 2;15(7):e0235532. doi: 10.1371/journal.pone.0235532 (PMC7332064; doi:10.1371/journal.pone.0235532)

S2 Fig. (A) Restriction analysis of recovered plasmids pPICH-CEN1, 2, and 4. Plasmids were digested with *Not*I and analyzed on 1% agarose gel**.** All digested plasmids yielded a common 4.2 kb band that represents the *ADE3* gene. The sizes of the upper bands represent the sum of individual centromeres and other common vector sequences (CEN1 = 7.6 kb; CEN2 = 9.0 kb; CEN4 = 8.7 kb). The upper band on pPICH-CEN1-*Not*I represents a partial digestion. M: 1 kb Plus DNA Ladder (Thermo Fisher Scientific). **(B) Restriction analysis of pPICH-ADE3.** The plasmid was digested with *Not*I and analyzed on 1% agarose gel. The 4.2-kb band represents the *ADE3* gene, and the 2.2-kb band represents the selection marker and *E. coli* sequences. M: 1kb Ladder Plus (Sinapse Inc).

(A)

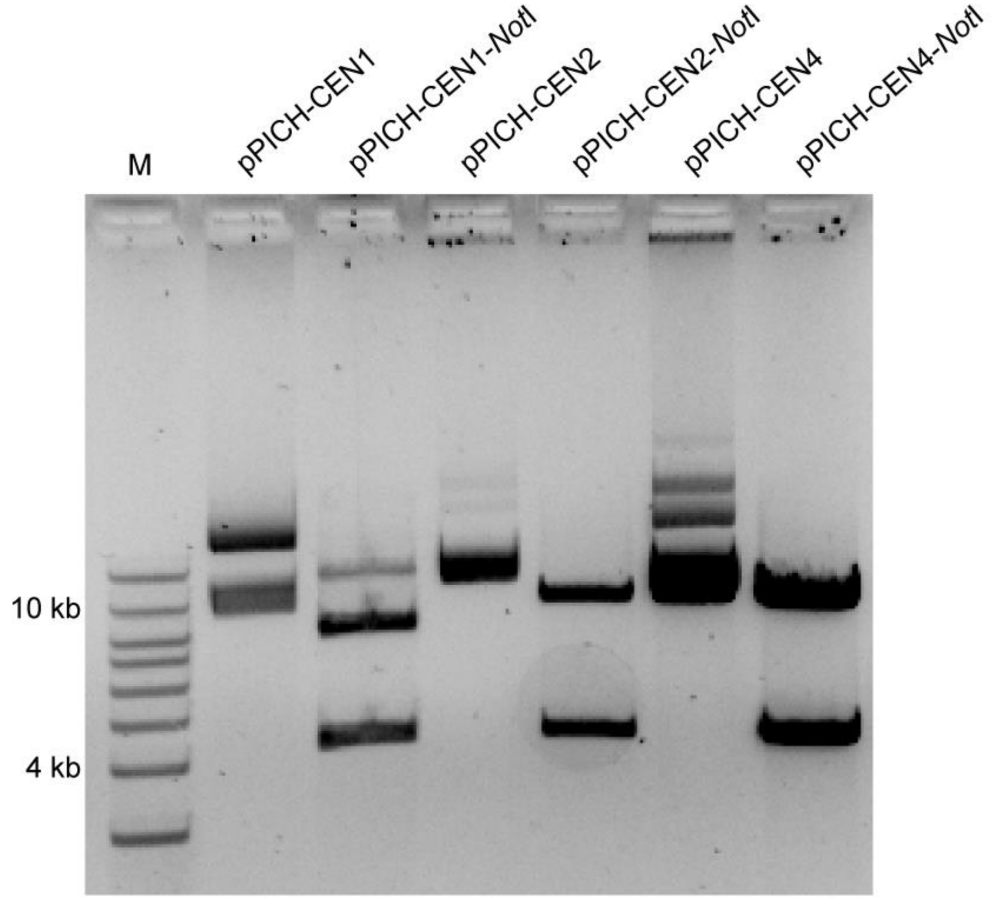

(B)

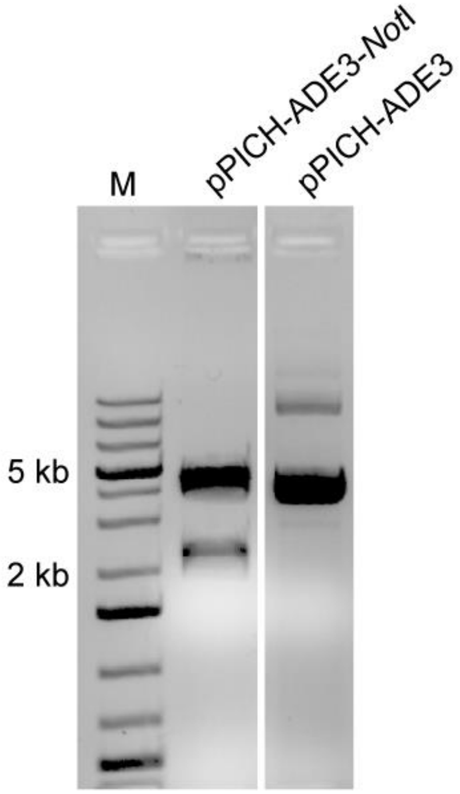

Supplement: S2 Fig — (A) Restriction analysis of recovered plasmids pPICH-CEN1, 2, and 4. Plasmids were digested with NotI and analyzed on 1% agarose gel. All digested plasmids yielded a common 4.2 kb band that represents the ADE3 gene. The sizes of the upper bands represent the sum of individual centromeres and other common vector sequences (CEN1 = 7.6 kb; CEN2 = 9.0 kb; CEN4 = 8.7 kb). The upper band on pPICH-CEN1-NotI represents a partial digestion. M: 1 kb Plus DNA Ladder (Thermo Fisher Scientific). (B) Restriction analysis of pPICH-ADE3. The plasmid was digested with NotI and analyzed on 1% agarose gel. The 4.2-kb band represents the ADE3 gene, and the 2.2-kb band represents the selection marker and E. coli sequences. M: 1kb Ladder Plus (Sinapse Inc). (PDF) [file pone.0235532.s002.pdf]
