# Supplementary material for: Construction and characterization of centromeric plasmids for Komagataella phaffii using a color-based plasmid stability assay
Source: PLoS One. 2020 Jul 2;15(7):e0235532. doi: 10.1371/journal.pone.0235532 (PMC7332064; doi:10.1371/journal.pone.0235532)
Supplement: S3 Fig — Plasmids pPICH-CEN1, pPICH-CEN2, and pPICH-CEN4. Images were generated using SnapGene 5.0.7. (PDF) [file pone.0235532.s003.pdf]

S3 Fig. Maps of the centromeric plasmids constructed in this study. Plasmids pPICH-CEN1, pPICH-CEN2, and pPICH-CEN4. Images were generated using SnapGene 5.0.7.

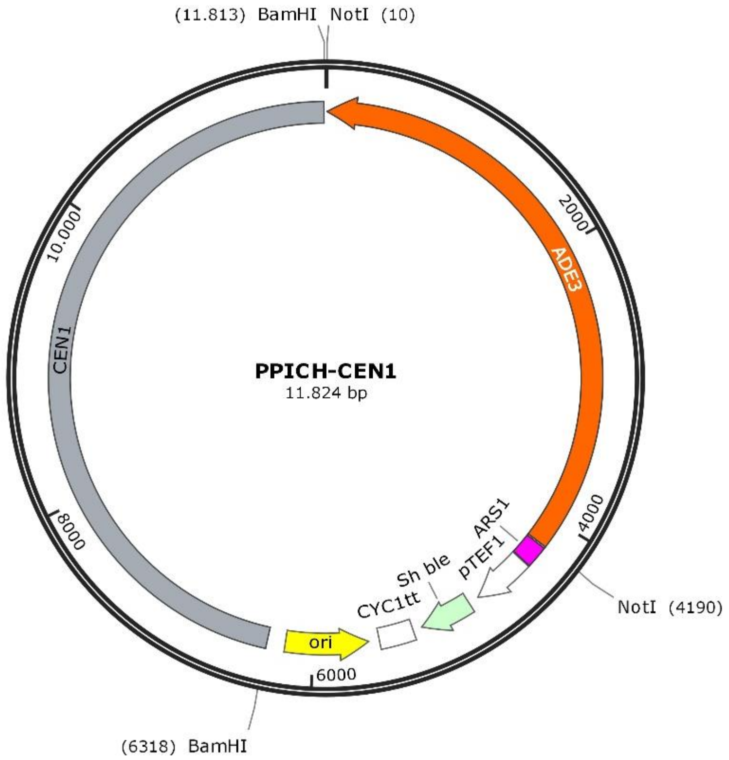

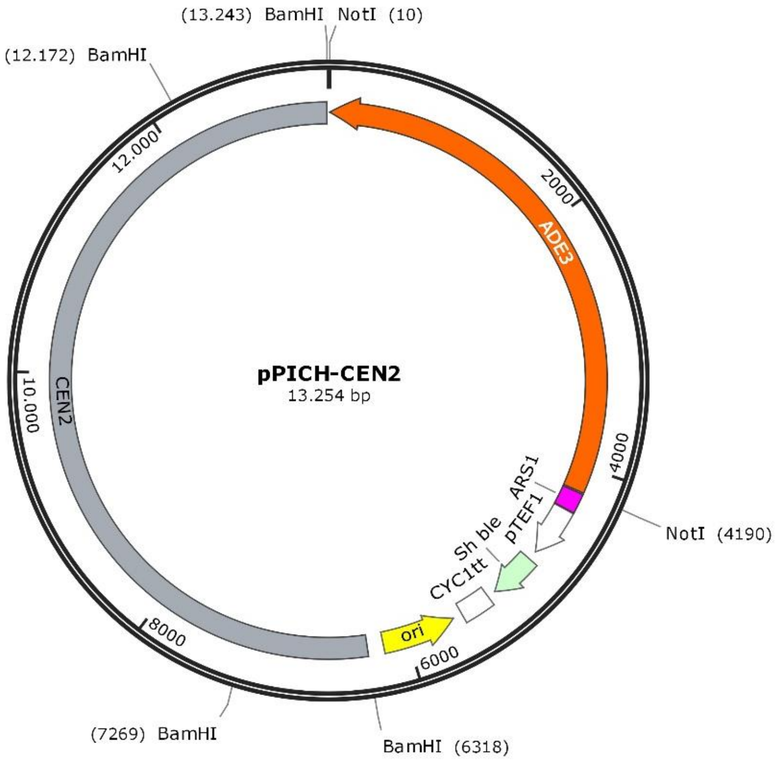

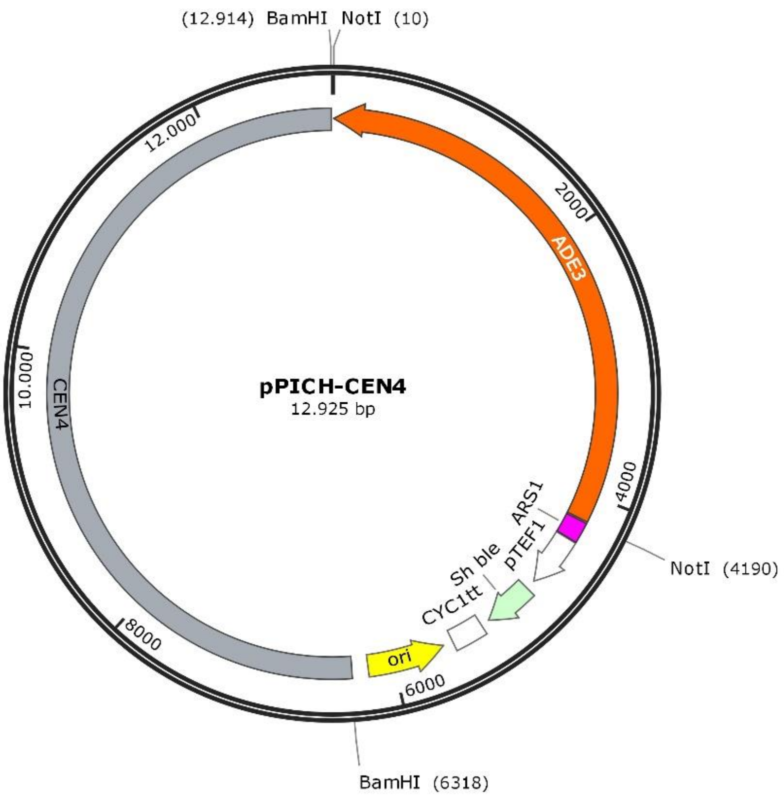
(12.914) BamHI NotI (10)
12.000
2000
ADE3
pPICH-CEN4
12.925 bp
10.000
CEN4
4000
ARS1
pTEF1
Sh ble
CYC1tt
NotI (4190)
8000
ori
6000
BamHI (6318)
