# Supplementary material for: Construction and characterization of centromeric plasmids for Komagataella phaffii using a color-based plasmid stability assay
Source: PLoS One. 2020 Jul 2;15(7):e0235532. doi: 10.1371/journal.pone.0235532 (PMC7332064; doi:10.1371/journal.pone.0235532)
Supplement: S1 Table — (PDF) [file pone.0235532.s004.pdf]

S1 Table. Transformation efficiencies obtained in 3 transformation events with pPICH-ADE3 and pPICH-CEN1-4.

**Plasmid**	**Transformation efficiency (CFU/μg DNA) ± standard deviation (n=3)**
pPICH-ADE3	825 ± 104
pPICH-CEN1	98 ± 39
pPICH-CEN2	60 ± 72
pPICH-CEN4	10 ± 8
